# Screening for Elder Mistreatment Risk in Caregivers: Clinician Perspectives on Barriers, Feasibility, & Response

**DOI:** 10.1093/geroni/igaf122.228

**Published:** 2025-12-31

**Authors:** Kelly Marnfeldt, Eleanor Batista-Malat, Zachary Gassoumis, Bonnie Olsen, Laura Mosqueda, Alexis Coulourides Kogan

**Affiliations:** University of Southern California Leonard Davis School of Gerontology, Los Angeles, California, United States; University of Southern California, Los Angeles, California, United States; UTHealth Houston, Houston, Texas, United States; University of Southern California, Los Angeles, California, United States; University of Southern California, Pasadena, California, United States; Keck Medicine of USC, Los Angeles, California, United States

## Abstract

Elder mistreatment (EM) risk screening in primary care presents challenges, particularly when aimed at assessing caregivers of people living with dementia. This secondary qualitative analysis examines how primary care clinicians conceptualize elder mistreatment, the feasibility of screening caregivers, and the perceived barriers and facilitators to screening implementation. Data were drawn from five focus groups (N = 38) conducted with multidisciplinary personnel at general and geriatric primary care clinics. Using Braun and Clarke’s reflexive thematic analysis, we analyzed focus group discussions to explore attitudes toward elder mistreatment screening among caregivers of patients with dementia, its practicality in primary care workflows, and related concerns. Participants viewed EM screening as feasible under certain conditions but raised concerns about time constraints, difficulty in dementia caregiver identification, and cultural and linguistic barriers. Some worried that screening might feel accusatory rather than supportive, emphasizing the need for careful framing. Respondents struggled with defining who qualifies as a caregiver, particularly when caregivers were not included in medical records or were absent during visits. While many saw screening as a potential opportunity to assess caregiver needs and connect them with resources, they noted that without clear implementation strategies and resources, feasibility remained uncertain. Effective implementation of dementia caregiver-focused EM risk screening requires clinician and site buy-in, cultural responsiveness, and framing that reassures rather than alienates caregivers. Findings highlight the importance of embedding screening within broader caregiver support efforts and ensuring clinicians feel equipped to navigate sensitive conversations.

